# Resistance Exercise Minimal Dose Strategies for Increasing Muscle Strength in the General Population: an Overview

**DOI:** 10.1007/s40279-024-02009-0

**Published:** 2024-03-20

**Authors:** James L. Nuzzo, Matheus D. Pinto, Benjamin J. C. Kirk, Kazunori Nosaka

**Affiliations:** Centre for Human Performance, School of Medical and Health Sciences, Edith Cowan University, 270 Joondalup Drive, Joondalup, WA 6027 Australia

## Abstract

Many individuals do not participate in resistance exercise, with perceived lack of time being a key barrier. Minimal dose strategies, which generally reduce weekly exercise volumes to less than recommended guidelines, might improve muscle strength with minimal time investment. However, minimal dose strategies and their effects on muscle strength are still unclear. Here our aims are to define and characterize minimal dose resistance exercise strategies and summarize their effects on muscle strength in individuals who are not currently engaged in resistance exercise. The minimal dose strategies overviewed were: “Weekend Warrior,” single-set resistance exercise, resistance exercise “snacking,” practicing the strength test, and eccentric minimal doses. “Weekend Warrior,” which minimizes training frequency, is resistance exercise performed in one weekly session. Single-set resistance exercise, which minimizes set number and session duration, is one set of multiple exercises performed multiple times per week. “Snacks,” which minimize exercise number and session duration, are brief bouts (few minutes) of resistance exercise performed once or more daily. Practicing the strength test, which minimizes repetition number and session duration, is one maximal repetition performed in one or more sets, multiple days per week. Eccentric minimal doses, which eliminate or minimize concentric phase muscle actions, are low weekly volumes of submaximal or maximal eccentric-only repetitions. All approaches increase muscle strength, and some approaches improve other outcomes of health and fitness. “Weekend Warrior” and single-set resistance exercise are the approaches most strongly supported by current research, while snacking and eccentric minimal doses are emerging concepts with promising results. Public health programs can promote small volumes of resistance exercise as being better for muscle strength than no resistance exercise at all.

## Key Points

**Table:** 

Many individuals do not perform resistance exercise, with perceived lack of time a commonly cited barrier to participation.
Minimal dose resistance exercise, which is resistance exercise that generally does not meet recommended guidelines and involves minimal time investment, warrants consideration for future health promotion efforts.
We define and overview evidence for five minimal dose strategies: “Weekend Warrior,” single-set resistance exercise, resistance exercise “snacking,” practicing the strength test, and minimal eccentric resistance exercise.
Minimal dose strategies generally improve muscle strength and some other fitness outcomes; thus, they can be recommended to individuals who do not perform resistance exercise.

## Muscle Strength and Current Guidelines for Resistance Exercise

Muscle strength refers to the maximal force that an individual can generate from their muscle voluntarily. Muscle strength decreases with aging [1]; thus, its maintenance or improvement is important for being able to meet the demands of daily life. Lower muscle strength correlates with or causes poor health outcomes including increased mortality risk, increased risk of falls, and reduced ability to perform activities of daily living [1–3].

The most effective way for someone to improve, maintain, or restore their muscle strength is by participating regularly in resistance exercise. Resistance exercise is planned and repeated muscle actions against external resistance or one’s body weight. Regular participation in resistance exercise improves physical and mental health and is associated with reduced mortality [4–10]. Public health bodies recommend that individuals participate in resistance exercise or other muscle-strengthening activities (e.g., heavy gardening or carrying heavy loads) ≥ 2 days per week [11]. 

Professional exercise science organizations have also published their own guidelines for resistance exercise participation (Table 1). The American College of Sports Medicine (ACSM) has published guidelines for healthy adults [4, 5] and older adults [12] as well as individuals with diabetes [13]. The National Strength and Conditioning Association (NSCA) has published guidelines for healthy youth [14] and older adults [15]. Exercise and Sports Science Australia (ESSA) has published guidelines for individuals with cancer [16], chronic heart failure [17], chronic kidney disease [18], chronic obstructive pulmonary disease [19], diabetes [20], hypertension [21], multiple sclerosis [22], obesity [23], osteoporosis [24], peripheral arterial disease [25], and spinal cord injury [26]. Overall, these guidelines recommend that individuals participate in two or three resistance exercise sessions per week, with each session consisting of eight to ten exercises that target large muscle groups. The guidelines also recommend that each exercise be performed for one to three sets of 8–15 eccentric–concentric repetitions using a moderate movement speed (1–2 s concentric and 1–2 s eccentric) and interset rests of 1–3 min.

**Table 1:** Professional organizations’ population-specific guidelines for resistance exercise

Organization	Population	Equipment	Session volume							Frequency (days/week)
			Exercises	Sets	Reps	Intensity (%1RM)	Rest (min)	Velocity	Duration (min)	
ACSM [4, 5]	Adults	FW, weight machines	NR, major muscles	1–4	8–20	≥ 60%	2–3	S, M	NR	2–3 NC
ACSM [12]	Older adults	NR	8–10, major muscles	NR	8–12	RPE: 5–8 (0–10 scale)	NR	NR	NR	≥ 2
NSCA [15]	Older adults	FW, weight machines	8–10, major muscles	1–3	6–15	70–85%	1.5–3	NR	NR	2–3 NC
NSCA [14]	Youth	FW, weight machines, bands, BW	NR, upper-, lower-body	1–3	6–15	50–85%	1–3	M	NR	2–3 NC
ESSA [16]	Cancer	FW, weight machines, bands, BW	NR, major or injured muscles	NR	NR	Moderate to high	NR	NR	NR	≥ 2 NC
ACSM and partners [151]	Cancer	NR	NR	≥ 2	8–15	≥ 60%	NR	NR	NR	≥ 2
ESSA [17]	Chronic heart failure	Weights, bands, BW	NR	NR	NR	RPE: 11–15 (0–20 scale)	NR	NR	20–60	2–3
ESSA [18]	Chronic kidney disease	FW, weight machines, weight cuffs, bands	NR	1 to fatigue	10–15	60–70%	NR	NR	NR	2 NC
ESSA [19]	COPD	FW, BW, machines	8–10, major muscles	NR	10–15	30–40% upper body, 50–60% lower body	NR	NR	10–20	2–3
ACSM [13]	Diabetes	FW, BW, machines, bands	5–10, major muscles	1–3	10–15	50–85%	NR	NR	NR	2–3 NC
ESSA [20]	Diabetes	NR	8–10, large muscles	2–4	8–10	Moderate to vigorous	NR	NR	60	≥ 2
ESSA [21]	Hypertension	FW, weight machines, bands, BW	8–10, major muscles	≥ 1 to fatigue	8–12	Sets to “substantial fatigue”	NR	NR	NR	≥ 2 NC
ESSA [22]	Multiple sclerosis	FW, weight machines, bands, BW	5–10, whole-body, lower-body focus	1–4	8–15	70–80%	2–4	NR	NR	2–3 NC
ESSA [23]	Obesity	FW, weight machines	NR	NR	NR	> 75%	NR	NR	NR	3
ESSA [24]	Osteoporosis	FW, weight machines	8, major muscles attached to hip, spine	2–3	8	80–85% RPE: ≥ 16 (0–20 scale)	NR	Some F	NR	2
ESSA [25]	Peripheral arterial disease	NR	6–8, large muscles, lower-body focus	2–3	8–12	60–80%	NR	Some F	NR	2 NC
ESSA [26]	Spinal cord injury	FW, weight machines, bands	≥ 4–5, major muscles, emphasize upper limb	3	8–12	60–70%	NR	NR	NR	≥ 2

*ACSM* American College of Sports Medicine, *BW* body weight, *COPD* chronic obstructive pulmonary disease, *ESSA* Exercise Sports Science Australia, *F* fast, *FW* free weights, *M* moderate, *NC* nonconsecutive days, *NR* not reported, *NSCA* National Strength and Conditioning Association, *RPE* rating of perceived exertion, *S* slow, *1RM* one repetition maximum

Most individuals do not meet recommended guidelines for participation in resistance exercise or muscle-strengthening activities [27–29]. A recent review of population-level surveys revealed that approximately 80% of individuals do not meet recommended guidelines for muscle-strengthening activities [27]. Moreover, approximately 58% of individuals do not participate in *any* muscle-strengthening activities [28], and 80% of individuals *never* participate in free weight or weight machine resistance exercise [29]. In Table 2, we summarize results from ten studies that have reported proportions of populations that do not participate in any resistance exercise or muscle-strengthening activities.

**Table 2:** Summary of studies that have reported proportions of populations that do not participate in *any* resistance exercise or muscle-strengthening activities

Reference	Country	Year	Sample (*n*)	Outcome assessed	% of respondents confirming no participation in resistance exercise	
					Men	Women
Humphries et al. [152]	AUS	2010	1230	No gym-based resistance training in past week	87.2	85.2
Humphries et al. [29]	AUS	2018	1237	No current strength training using machines, free weights	77.6	82.3
Scholes et al. [153]	ENG	2012	8291	No MSA in past month	49	56
Livingstone et al. [154]	IRE	2001	1379	No “exercise with weights” in past year	90.3	94.3
Firebaugh [155]	USA	1989	33,360	No “weight lifting” in past 2 weeks	69.2–96.8	90–99.3
Eaton et al. [156]	USA	1994	33,428	No “weight lifting” that works up sweat ≥ one time/week	94.2–99.8	99.3–100
CDC [157]	USA	1996	~ 35,000	No “weight lifting” or other activity to increase strength in past 2 weeks	80	85.9
Powell et al. [158]	USA	1998	5236	No “weightlifting” in past 30 days	69.8	87.6
Galuska et al. [159]	USA	2002	16,697	Did not “lift weights” in past month	80.5	92.3
Bennie et al. [28]	USA	2018	397,423	No MSA in past week	53.6	61.8

*AUS* Australia, *CDC* Centers for Disease Control and Prevention, *ENG* England, *IRE* Ireland, *MSA* muscle-strengthening activities, *USA* United States of America

Perceived lack of time is one of the most frequently cited barriers to exercise participation [30–37]. Perceived lack of time is also a reason why individuals do not adhere to exercise prescriptions and why some individuals discontinue exercise [38–40]. Given that lack of time is a key obstacle to exercise participation, and many individuals do not participate in any resistance exercise, alternative strategies for facilitating participation appear warranted. Minimal dose resistance exercise is one potential strategy.

## Minimal Dose Resistance Exercise

We define minimal dose resistance exercise as resistance exercise that does not meet guidelines recommended by professional exercise organizations but that still has the potential to improve muscle strength. Typically, minimal dose resistance exercise prescriptions will have lower weekly training volumes compared with prescriptions that are consistent with recommended guidelines. A resistance exercise dose can be made more minimal than current prescription guidelines by reducing exercise frequency, session duration, and/or volume compared with recommended guidelines (Tables 3, 4). The relative load used (i.e., “intensity”), proximity to failure, and the muscle contraction type performed during exercise are also important variables of exercise prescriptions that warrant consideration in minimal dose prescriptions.

**Table 3:** Summary of minimal dose approaches to resistance exercise dosing

Approach name	Variable of exercise prescription that is minimized	Description
“Weekend Warrior”	Frequency of exercise sessions each week	Total resistance exercise volume for the week is completed in one (or perhaps two) sessions. The exercise volume, which typically consists of multiple sets of various exercise at submaximal loads, may or may not meet current recommendations for resistance exercise volume
Single-set resistance exercise	Number of sets of exercise completed in each session	One exercise set for multiple exercises (eight to ten exercises) at submaximal loads is completed at a frequency of ≥ 2 days/week
Resistance exercise “snacks”	Duration of each exercise session	A low volume of resistance exercise that is performed once or more daily
Practicing the strength test	Number of exercises and repetitions completed in each session	One repetition per exercise set with a maximal resistance and repeating this for multiple sets. When performed daily, practicing the strength test is a resistance exercise “snack”
Minimal dose eccentric resistance exercise	Number of concentric muscle actions completed in each session is zero and number of eccentric muscle actions in each session is minimal	A low volume of submaximal or maximal resistance exercise that involves eccentric-only repetitions. When performed daily, minimal dose eccentrics are a resistance exercise “snack”

**Table 4:** Impact of minimal dose resistance exercise strategies on weekly resistance exercise prescriptions compared with current recommended guidelines by programming variable

Minimal dose strategy	Variable of traditional resistance exercise prescription (Table 1)					
	Exercises	Sets	Repetitions	Intensity	Frequency	Time
	5–10 per session	1–4 per exercise	8–15 per set	50–85 % max	2–5 days/week	30–90 min per session
“Weekend Warrior” 8–10 exercises/session 1–3 sets/exercise 8–12 ECC-CON reps/set 60–80% max 1 day/week ≥ 45 min/session	↔	↔	↔	↔	↓	↔
Single-set resistance exercise 7–10 exercises/session 1 set/exercise 6–20 ECC-CON reps/set 60–85% max 2 or 3 days/week 30 min/session	↔	↔ ↓	↔	↔	↔	↔ ↓
Resistance exercise “snacks” 1–5 exercises/snack 1 set/snack ~ 12 ECC-CON reps/set ≤ 80% max 5–7 days/week 2–10 min × ≥ 2 per day	↓	↔	↔	↔	↑	↓
Practicing the strength test ≥ 1 exercise/session ≥ 1 set/exercise 1 rep/set 100% max 2–7 days/week ≤ 5 min/session	↔ ↓	↔ ↓	↓	↑	↑ ↔	↓
Eccentric minimal dose ≤ 5 exercises/session 1–5 sets/exercise ≤ 6 ECC-only reps/set 50–100% max 1–7 days/week ≤ 10 min/session	↔ ↓	↔ ↓	↓	↑ ↔	↔	↓

↓ Aspect of the minimal dose programs is lower compared with traditional resistance exercise prescriptions. ↑ Aspect of the minimal dose programs is higher compared with traditional resistance exercise prescriptions. ↔ Aspect of the minimal dose programs is similar to traditional resistance exercise prescriptions

Cells containing multiple arrows indicate the programming variable may or may not be modified in a minimal dose prescription compared with traditional prescriptions guidelines

*CON* concentric, *ECC* eccentric

One reason for examining the potential benefits of minimal dose approaches is to determine if current guidelines for resistance exercise or muscle-strengthening activities, and the public health messaging that promotes them, might require additional consideration. For example, though minimal resistance exercise doses might not meet recommended guidelines, they might still allow for individuals who are not partaking in any physical exercise to increase their muscle strength and perhaps obtain other health benefits associated with resistance exercise participation. Moreover, minimal participation in resistance exercise might act as a “gateway” or “stepping stone” to more frequent participation in the future, though this hypothesis is speculative.

## Aim of Paper

**Fig. 1:**
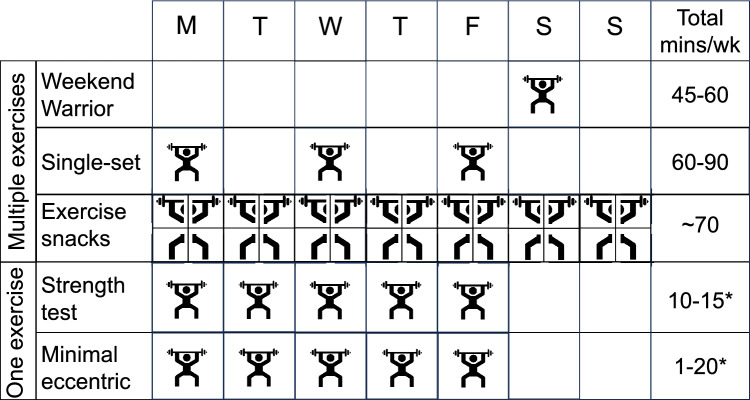
Visual representation of program variable characteristics of five minimal dose resistance exercise strategies: “Weekend Warrior,” single-set resistance exercise, resistance exercise “snacks,” practicing the strength test, and minimal dose eccentric resistance exercise. See Tables 3 and 4 for details on each minimal dose strategy. The graphic for resistance exercise "snacks" represents four different "snacks" completed on a given day. *Because practicing the strength test and minimal dose eccentrics have been studied mostly at the proof-of-concept stage, and related study interventions have usually only included only one exercise, the total weekly exercise time for those two approaches presented in the figure is only for one exercise rather than multiple exercises. *MVC* maximal voluntary contraction, *RM* repetition maximum

In recent years, exercise scientists have studied or advocated for minimal or time-efficient dose approaches to resistance exercise [41–48]. Previous discussions have typically centered around one or two minimal dose approaches. However, broad overviews of the multiplicity of minimal dose approaches, their definitions, and their evidence is lacking. Therefore, the aims of the current overview are to define and characterize resistance exercise strategies that reflect the minimal dose concept and to summarize evidence of their impact on muscle strength (and other reported outcomes of health and fitness) in persons not currently engaged in resistance exercise. Five strategies were identified that fit the minimal dose concept: “Weekend Warrior,” single-set resistance exercise, resistance exercise “snacking,” practicing the strength test, and eccentric minimal doses (Fig. 1). We then discuss the implications and limitations of these minimal dose approaches and directions for future research.

## Methods

Due to the varying literature on minimal dose exercise, and because some study interventions might not have been conceived of or labeled as minimal dose at the time of publication, we chose an overview method rather than a systematic review method to best summarize the relevant literature [49, 50]. We have used similar nonsystematic approaches (e.g., “snowballing” searches) in several comprehensive literature reviews [27, 50–54]. Nevertheless, we acknowledge that some relevant studies could be missing from our discussion and that study quality was not assessed.

In general, we tried to limit our overview to studies in which participants had little or no recent history with resistance exercise (i.e., “untrained”). Nevertheless, we cite some studies that included participants with resistance exercise experience. We have done this in instances where, for example, data in untrained individuals were scarce or to illustrate the overall robustness or validity of a concept. Where feasible, we have noted resistance experience in the text and tables.

The distinction between studies in the current overview that might be considered “proof-of-concept” versus “ecologically valid” is also important. In the current overview, a proof-of-concept study refers to one in which a basic experimental model was used to test whether a certain minimal volume of resistance exercise increases muscle strength. An example of this is a study that uses a model of eccentric-only repetitions of the elbow flexor muscles performed on an isokinetic dynamometer in a laboratory and under supervision. One reason that such a study can be classified as proof-of-concept is that, unless one is undertaking a rehabilitation program for a specific body muscle group, the goal of most resistance exercise prescriptions is to increase muscle strength for multiple muscle groups. Therefore, when discussing minimal dose approaches, one can reference results from studies whose exercise programs are broadly reflective of “real-world” versions of such programs. For example, “Weekend Warrior” and single-set resistance exercise interventions often include multiple exercises that target major muscle groups. On the other hand, practicing the strength test and eccentric minimal doses are still at the proof-of-concept stage, and their ecological validity might require demonstration in future research. In Table 4, when comparing the five minimal dose approaches to traditional resistance exercise guidelines, we mostly assumed the mature or real-world versions of these minimal dose strategies rather than their proof-of-concept forms (e.g., multiple muscle groups targeted rather than a single muscle group). Also, in constructing the concept of a minimal dose in the current paper, we refer to the weekly dose of resistance exercise. That is, we consider strategies that can minimize exercise time, not just from the perspective of a single exercise session, but across an entire week.

## “Weekend Warriors”

“Weekend Warrior” is a phrase used to describe an individual who performs all of their exercise within one (or perhaps two) exercise sessions each week [55]. The exercise prescription variable that is minimized with the “Weekend Warrior” approach is session frequency. Some “Weekend Warrior” doses meet recommended exercise guidelines, whereas others do not [56]. Thus, for the current review, we consider the “Weekend Warrior” approach a potential minimal dose strategy. Approximately 1–3% of adults in the USA are “Weekend Warriors” [55].

In the first study on “Weekend Warriors,” which was not exclusive to participation in resistance exercise, Lee et al. [57] found that men classified as “Weekend Warriors” at baseline had a lower risk of dying over a 9-year follow-up period than sedentary men. Subsequent studies confirmed that 1–2 days per week of physical exercise, not exclusive to resistance exercise, reduced mortality risk [56–60] and incidence of cardiovascular disease [61] compared with no exercise.

The impact of frequency of resistance exercise on muscle size and strength has also been examined in several studies and reviews of individuals with and without backgrounds in resistance exercise [62–64]. In the late 1980s and early 1990s, a series of studies on the extensor muscles of the cervical and lumbar spine illustrated that one session of resistance exercise per week, which involved only one exercise of eccentric–concentric repetitions, improved muscle strength of the targeted muscles in untrained [65–67] and trained individuals [68]. The findings provided proof of concept that total body resistance exercise programs might increase muscle strength when participation occurs only once per week. Thirty years of subsequent research has illustrated that this is the case. In individuals who are currently not undertaking resistance exercise, one session of resistance exercise per week, consisting of four or more exercises of eccentric–concentric repetitions per session, improves muscle strength and other measures of physical fitness compared with no exercise (Table 5).

**Table 5:** “Weekend Warrior”—summary of results from studies that examined changes in muscle strength after resistance exercise programs that were completed 1 day per week and comprised of coupled eccentric–concentric repetitions for ≥ four exercises per session

Reference	Group	Sample				Session					Week			Program	
		RE past	*n*	Sex	Age (years)	Exercise no.	Set no.	Rep no.	Intensity (%max)	Time (min)	FREQ (days/week)	Set no.	Rep no	Duration (weeks)	Strength (% $\Delta$)
Taafee et al. [160]	EX1	No	14	MW	69	8	3	8	80	NR	1	24	192	24	21–74^b^
	CON	No	12	MW	69	0	0	0	0	0	0	0	0	24	0–10^b^
McLester et al. [161]	EX1	Yes	9	MW	25	9	3	Fail	80	NR	1	27	~ 216	12	20–25^b^
	CON	n/a	n/a	n/a	n/a	n/a	n/a	n/a	n/a	n/a	n/a	n/a	n/a	12	n/a
Bates et al. [162]	EX1	No	110	MW	68	14	2	8–10	NR	60	1	28	224–280	10	NR
	CON	n/a	n/a	n/a	n/a	n/a	n/a	n/a	n/a	n/a	n/a	n/a	n/a	10	n/a
DiFrancisco-Donoghue et al. [163]	EX1	No	9	MW	73	9	1	Fail	75	NR	1	9	~ 110	9	23–44^b^
	CON	n/a	n/a	n/a	n/a	n/a	n/a	n/a	n/a	n/a	n/a	n/a	n/a	9	n/a
Liu-Ambrose et al. [164]	EX1	No	54	W	70	10	2	6–8	6–8RM	60	1	20	120–160	52	19^b^
	CON	No	n/a	n/a	n/a	n/a	n/a	n/a	n/a	n/a	n/a	n/a	n/a	n/a	n/a
Farinatti et al. [165]	EX1	No	10	W	72	10	1	10	70	NR	1	10	100	16	40–57^b^
	CON	No	10	W	68	0	0	0	0	0	0	0	0	16	2–4^b^
Sousa et al. [166]	EX1	NR	16	M	69	7	3	8–12	65–75	NR	1	21	168–252	32	↑ NR^b^
	CON	NR	17	M	67	0	0	0	0	0	0	0	0	32	NR
Orsatti et al. [167]	EX1	No	9	W	57	10	3	8–12	60–80	50	1	30	240–360	16	20 ^b^
	CON	n/a	n/a	n/a	n/a	n/a	n/a	n/a	n/a	n/a	n/a	n/a	n/a	16	n/a
Gentil et al. [168]	EX1	No	15	M	23	8	3	Fail	8–12RM	NR	1	24	192–288	10	7^c^
	CON	n/a	n/a	n/a	n/a	n/a	n/a	n/a	n/a	n/a	n/a	n/a	n/a	10	n/a
Turpela et al. [169]	EX1	No	24	MW	70	NR	2–5	4–12	30–90	NR	1	NR	NR	24	~ 6^a^
	CON	No	20	MW	69	0	0	0	0	0	0	0	0	24	~ − 2^a^
Richardson et al. [170, 171]	EX1	No	10	MW	66	8	3	14	40	NR	1	24	336	10	8–17^b^
	EX2	No	10	MW	67	8	3	7	80	n/a	1	24	168	10	24–30^b^
Moraes et al. [172], dos Santos et al. [173]	EX1	No	12	W	55	5	3	Fail	8–12RM	35	1	15	120–180	8	20–34^d^
	CON	No	13	W	54	0	0	0	0	0	0	0	0	8	− 4 to 2^d^
Geneen et al. [174]	EX1	No	7	MW	53	6	3	8	80	60	1	18	144	12	31–44^a^
	CON	n/a	n/a	n/a	n/a	n/a	n/a	n/a	n/a	n/a	n/a	n/a	n/a	n/a	n/a
Lee and Yoo [175]	EX1	No	15	M	44	6	3	12	BW	45	1	18	216	20	6^a^
	CON	No	15	M	49	0	0	0	0	0	0	0	0	20	10^a^

Other outcomes that improved in some of the above studies after once-weekly resistance exercise included: muscle endurance [162, 166, 170], muscle size or body composition [168, 174, 175], blood pressure [161], up-and-go time [162, 165, 169, 170], sit-to-stand repetitions or time [160, 166, 170], flexibility [162], Stroop test performance [164], and quality of life [172, 175]. Other studies reported no change in body composition [161, 167, 169], stair climb performance [169], or flexibility [170]

*BW* body weight resistance, *CON* control group (no exercise), *EX* exercise group, *FREQ* frequency, *M* men, *n/a* not applicable, *NR* not reported, *RE* resistance exercise, *REPS* repetitions per exercise set, *RM* repetition maximum, *W* women

^a^Isometric strength test

^b^One repetition maximum concentric strength test

^c^Isokinetic concentric strength test

^d^≥ 5 repetition maximum concentric strength test

Literature reviews have indicated more frequent participation in resistance exercise causes greater increases in muscle size and strength (i.e., dose–response relationship) [62–64]. Nevertheless, differences in gains in muscle size and strength from different exercise frequencies disappear when exercise volume is equated between conditions [62–64]. Thus, performing one session of resistance exercise per week increases muscle size and strength, and this increase equals that which occurs with greater exercise frequencies when exercise volume is equated throughout the week.

Overall, the current evidence suggests that improvements in muscle strength can occur with resistance exercise that is completed only one day per week. The “Weekend Warrior” approach to resistance exercise can be advocated as a minimal dose approach for those who are currently not participating in resistance exercise. It is an approach most appropriate for individuals who have a particular day of the week when they have extended time available for exercise.

## Single-Set Resistance Exercise

Single-set resistance exercise involves performing one set of multiple exercises (usually seven to ten) in an exercise session with multiple sessions occurring each week. The variable of the exercise prescription that is minimized with single-set resistance exercise is the number of sets. The reduced number of sets then reduces session volume and duration compared with resistance exercise programs that consist of multiple sets for each exercise. We acknowledge that some guidelines presented in Table 1 list one set of resistance exercise at the lower end of the set range. However, because some guidelines recommend two or more exercise sets, and because one set of exercise is an approach that will reduce exercise session duration, we consider it a minimal dose approach.

The topic of whether one set and multiple sets of resistance exercise produce equal gains in muscle size and strength in individuals with varying levels of resistance exercise experience has been reviewed multiple times [69–72] and has been a point of contention for many years [73, 74]. Here, our focus is on whether one set of exercise causes within-group improvements in muscle strength and whether these improvements are greater than observed in control groups who do not perform resistance exercise. In Table 6, we summarize evidence showing that resistance exercise programs consisting of one set of coupled eccentric–concentric repetitions for four or more exercises per session (≥ 2 sessions per week) improve muscle strength and other markers of physical fitness in both untrained and trained individuals. Few studies included control groups for comparison, but the strength gains observed in the resistance exercise groups (~ 20%) were greater than those typically observed in control groups who do not participate in resistance exercise. Thus, single-set resistance exercise can be advocated as an effective minimal dose strategy for individuals who are currently not participating in resistance exercise.

**Table 6:** Single-set resistance exercise—summary of results from select studies that examined changes in muscle strength after resistance exercise programs completed ≥ 2 days per week and comprised of 1 set of coupled eccentric–concentric repetitions for ≥ four exercises per session

Reference	Group	Sample				Session					Week			Program	
		RE past	*n*	Sex	Age (years)	Exercise no.	Set no.	Rep no.	Intensity (%max)	Time (min)	FREQ (days/week)	Set no.	Rep no.	Duration (weeks)	Strength (% $\Delta$)
Capen [176]	EX1	NR	52	M	NR	5	1	8–15	8–15RM	NR	3	15	120	12	18^a^
	CON	n/a	n/a	n/a	n/a	n/a	n/a	n/a	n/a	n/a	n/a	n/a	n/a	12	n/a
Kramer et al. [177]	EX1	Yes	16	M	20	4	1	Fail	8–12RM	NR	3	12	96–144	14	12^b^
	CON	n/a	n/a	n/a	n/a	n/a	n/a	n/a	n/a	n/a	n/a	n/a	n/a	14	n/a
Hass et al. [178]	EX1	Yes	21	MW	40	9	1	Fail	8–12RM	NR	3	27	216–324	13	6–13^ab^
	CON	n/a	n/a	n/a	n/a	n/a	n/a	n/a	n/a	n/a	n/a	n/a	n/a	13	n/a
Schlumberger et al. [179]	EX1	Yes	9	W	29	7	1	6–9	6–9RM	NR	2	14	84–126	6	4–6^b^
	CON	Yes	9	W	25	0	0	0	0	0	0	0	0	6	− 3 to 0^b^
Rhea et al. [180]	EX1	Yes	8	M	22	7	1	4–10	4–10RM	60	3	21	84–210	12	20–26^b^
	CON	n/a	n/a	n/a	n/a	n/a	n/a	n/a	n/a	n/a	n/a	n/a	n/a	12	n/a
Vincent et al. [181]	EX1	No	24	MW	67	13	1	13	50	~ 30	3	39	507	24	17^b^
	EX2	No	22	MW	67	13	1	8	80	~ 30	3	39	312	24	18^b^
	CON	No	16	MW	71	0	0	0	0	0	0	0	0	24	− 9^b^
Galvão and Taaffe [182]	EX1	No	12	MW	69	7	1	Fail	8RM	NR	2	14	112	20	3–15 ^abc^
	CON	n/a	n/a	n/a	n/a	n/a	n/a	n/a	n/a	n/a	n/a	n/a	n/a	20	n/a
Rønnestad et al. [183]	EX1	No	10	M	26	8	1	7–10	7–10RM	NR	3	24	168–240	11	2–25^bc^
	EX2	No	11	M	26	8	3	7–10	7–10RM	NR	3	72	504–720	11	8–40^bc^
Baker et al. [184]	EX1	Yes	8	M	20	9	1	Fail	85	n/a	3	27	~ 160	8	22^b^
	CON	n/a	n/a	n/a	n/a	n/a	n/a	n/a	n/a	n/a	n/a	n/a	n/a	8	n/a
Radaelli et al. [185]	EX1	No	11	W	65	10	1	10–20	10–20RM	NR	2	20	200–400	13	14–32^ab^
	CON	n/a	n/a	n/a	n/a	n/a	n/a	n/a	n/a	n/a	n/a	n/a	n/a	13	n/a
Radaelli et al. [186]	EX1	No	11	W	64	10	1	6–20	6–20RM	NR	2	20	120–400	20	12–39^ab^
	CON	n/a	n/a	n/a	n/a	n/a	n/a	n/a	n/a	n/a	n/a	n/a	n/a	20	n/a
Abrahin et al. [187]	EX1	Yes	15	W	67	5	1	Fail	8–12RM	20	2	10	80–120	12	38–79^d^
	CON	n/a	n/a	n/a	n/a	n/a	n/a	n/a	n/a	n/a	n/a	n/a	n/a	12	n/a
Radaelli et al. [188]	EX1	Yes	12	W	84	9	1	8–12	8–12RM	NR	3	27	216–324	24	13–22^d^
	CON	n/a	n/a	n/a	n/a	n/a	n/a	n/a	n/a	n/a	n/a	n/a	n/a	24	n/a
Ribeiro et al. [189]	EX1	No	15	W	66	8	1	10–15	10–15RM	NR	3	24	240–360	12	16–20^b^
	CON	n/a	n/a	n/a	n/a	n/a	n/a	n/a	n/a	n/a	n/a	n/a	n/a	12	n/a
Schoenfeld et al. [190]	EX1	Yes	11	M	24	7	1	8–12	8–12RM	NR	3	21	168–252	8	10–18^b^
	CON	n/a	n/a	n/a	n/a	n/a	n/a	n/a	n/a	n/a	n/a	n/a	n/a	8	n/a

Other outcomes that improved in some of the above studies after single-set resistance exercise included: muscle endurance [178, 181, 190], muscle size or body composition [178, 183–186, 188], gait speed [182], stair climbing ability [181, 182], sit-to-stand repetitions and time [182, 187], and maximal inspiratory and expiratory pressures [187]

*CON* control group (no exercise), *FREQ* frequency, *EX* exercise group, *M* men, *n/a* not applicable, *NR* not reported, *RE* resistance exercise, *RM* repetition maximum, *W* women

^a^Isometric strength test

^b^One repetition maximum concentric strength test

^c^Isokinetic concentric strength test

^d^ ≥ 5 repetition maximum concentric strength test

## Daily Resistance Exercise “Snacks”

A resistance exercise “snack” is a low volume of resistance exercise that is performed once or more daily, often multiple days per week. Islam et al. [75] defined an exercise snack as “isolated ≤ 1-min bouts of vigorous exercise performed periodically throughout the day.” “Snacking” was initially used as an exercise prescription method to improve cardiometabolic health [76], but the technique has expanded to include resistance exercise prescriptions. The variable of the exercise prescription that is minimized with exercise snacks is session duration, and this is accomplished by reducing the number of exercises or sets compared with more traditional resistance exercise prescriptions. The frequency of exercise is also higher with snacks compared with more traditional approaches, and snacks have typically involved sets of eccentric–concentric repetitions [45, 46, 77, 78].

At least three studies have examined resistance exercise snacking programs in healthy adults without a recent history of resistance exercise (Table 7). Kowalsky et al. [45] examined daily resistance exercise snacks over a 1-week period on measures of muscular discomfort and sleepiness among 24 university students. Each day, participants completed eight different exercises as eight separate snacks distributed across the day. Each snack consisted of two sets of 15 repetitions of the following exercises: chair stands, desk/table pushups, alternating lunges, calf raises, biceps curls, lateral rows, upright rows, and deadlifts with a resistance band. The same participants also completed a control condition in which they did not complete the exercise snacks. The results revealed that participants experienced lower muscular discomfort and reduced daytime sleepiness during the week they completed the exercise versus the control week when they did not complete the exercise.

**Table 7:** Daily resistance exercise “snacks”—summary of results from studies that examined changes in muscle strength in healthy adults [45, 46, 77] and patient groups [79–83] after resistance exercise programs in which multiple, low volume exercise sessions (i.e., “snacks”) were completed 5 or 7 days per week

Reference	Group	Sample				Session					Week			Program	
		RE past	*n*	Sex	Age (years)	Exercise no.	Set no.	Rep no.	Intensity (%max)	Time (min)	FREQ (days/week)	Set no.	Rep no.	Duration (weeks)	Strength (% $\Delta$)
Kowalsky et al. [45]	EX1	NR	24	MW	23	1	2	15	BW, bands	3–5	8 snacks/day 7 days/week	112	1680	1	NR
	CON	n/a	n/a	n/a	n/a	n/a	n/a	n/a	n/a	n/a	n/a	n/a	n/a	n/a	n/a
Perkin et al. [46]	EX1	No	10	MW	70	5	1	1 min	BW	8	2 snacks/day 7 days/week	70	> 1000	4	5^b^
	CON	No	10	MW	70	0	0	0	0	0	0	0	0	4	− 2^b^
Fyfe et al. [77]	EX1	No	9	MW	70	5	1	1 min	BW	9	1 snack/day 7 days/week	35	> 1000	4	NR
	EX2	No	10	MW	69	5	1	1 min	BW	9	2 snacks/day 7 days/week	70	> 1000	4	NR
	EX3	No	9	MW	70	5	1	1 min	BW	9	3 snacks/day 7 days/week	105	> 1000	4	NR
	CON	No	10	MW	70	0	0	0	0	0	0	0	0	4	NR
Western et al. [83]	EX1	No	21	MW	78	5	1	1 min	BW	9	2 snacks/day 7 days/week	70	NR	4	NR
	CON	n/a	n/a	n/a	n/a	n/a	n/a	n/a	n/a	n/a	n/a	n/a	n/a	n/a	n/a
Andersen et al. [79, 80], Lidegaard et al. [82], Jay et al. [81]	EX1	No	63	MW	44	1 LR	1	Fail	8–12RM	2	5	5	40–60	10	6^a^
	EX2	No	65	MW	42	1 LR	5–6	Fail	8–12RM	12	5	25–30	200–360	10	5^a^
	CON	No	64	MW	43	0	0	0	0	0	0	0	0	10	1^a^

Other outcomes that improved in some of the above studies included: muscle size or body composition [46], sit-to-stand repetitions and time [46, 77, 83], balance [77, 83], time sleepiness [45], and discomfort, pain, or pain-related symptoms [45, 79–82]

*BW* body weight, *CON* control group (no exercise), *EX* exercise group, *FREQ* frequency, *LR* lateral raise, *M* men, *n/a* not applicable, *NR* not reported, *RE* resistance exercise, *RM* repetition maximum, *W* women

^a^Isometric strength test

^b^One repetition maximum concentric strength test

Two studies have also examined daily resistance exercise snacks in healthy older adults without a recent history of resistance exercise [46, 77]. In one study [46], ten older adults completed home-based resistance exercise snacks twice per day for 28 days. One snack occurred in the morning; the other snack occurred in the evening. Each snack consisted of five exercises, and each of the five exercises was completed for as many repetitions as possible over a 1-min period. The exercises included: chair sit-to-stand, seated knee extension, standing knee bends, marching on the spot, and standing calf raises. A 1-min rest separated each exercise, culminating in 9 min of exercise. Participants who completed the exercise program showed greater improvements in the sit-to-stand test, leg press power, and thigh muscle cross-sectional area than participants who did not participate in the exercise program.

In another study [77], community-dwelling older adults completed a home-based resistance exercise snack program delivered remotely once, twice, or three times per day for 4 weeks. Each snack was 9 min, totaling 9, 18, and 27 min of exercise per day for the three groups, respectively. Adherence rates to the exercise programs were 97, 82, and 81%, respectively. The interventions did not cause significant improvements in sit-to-stand performance compared with control (no exercise). However, the interventions were generally rated as enjoyable and easy to perform, and 82% of exercise participants planned to continue the exercise program after the study. The authors concluded that resistance exercise snacks may be feasible for home-based resistance exercise for older adults when delivered and monitored remotely.

Finally, one study examined the acute postprandial glycemic responses to resistance exercise snacks [78]. In one testing session, study participants sat for 4 h without activity in the evening. In another session, they performed three minutes of resistance exercise every 30 min over the 4 h. The resistance exercises were chair squats, calf raises, and standing knee raises with straight leg hop extensions. The main finding was that the resistance exercise snacks reduced postprandial glucose and insulin responses compared with no exercise, which suggests that interrupting sitting with brief resistance exercise might have cardiometabolic health benefits, although longer-term snack training studies are needed to confirm this hypothesis.

In a series of studies, resistance exercise snacks completed 5 days per week (sometimes considered “daily” exercise) were prescribed to individuals with neck and shoulder pain [79–82]. Andersen et al. [79] submitted individuals with frequent neck/shoulder pain to 10 weeks of daily progressive resistance exercise. Only the lateral raise exercise with elastic tube resistance was completed in the exercise program. One group of participants performed 2 min of exercise daily, and another group performed 12 min of exercise daily. Compared with a control group who did not complete the exercise, participants in the exercise groups showed larger reductions in neck/shoulder pain and tenderness and greater improvements in muscle strength. The authors concluded that as little as 2 min of targeted daily progressive resistance exercise caused clinically meaningful reductions in pain and tenderness in adults with frequent neck/shoulder symptoms. In a similar study of individuals who had frequent neck/shoulder pain, Jay et al. [81] found that 10 weeks of resistance exercise with elastic tubes 2 or 12 min per day increased muscle strength and rate of force development more than no resistance exercise. Also, in 30 female office workers with chronic neck and shoulder pain, Lidegaard et al. [82] found that 2 min per day of resistance exercise with elastic tubes increased muscle strength and decreased pain intensity compared with no resistance exercise. Finally, in a study of 198 office workers who had frequent neck/shoulder pain, Andersen et al. [80] found that 10 weeks of resistance exercise with elastic tubes used for 2 or 12 min per day decreased headache frequency compared with no resistance exercise but did not impact headache intensity and duration.

Overall, resistance exercise snacking shows promise as a minimal dosing strategy for improving movement capacity in older adults [46, 77, 83] and reducing pain in patients with neck and shoulder pain [79–82]. Many of the reviewed studies utilized low-effort body weight exercises. Thus, these snacking approaches might be most applicable to older adults, patients, and individuals who dislike exercising due to perceived discomfort associated with exercise intensity. Future research can explore more thoroughly the impact of resistance exercise snacking on health and fitness in younger and healthier adults who are currently not partaking in resistance exercise.

## Practicing the Strength Test

Practicing the strength test involves performing one repetition per set with a maximal resistance and doing this for one or more sets within a given session. The variable of the exercise prescription that is minimized with practicing the strength test is the number of exercise repetitions. This then reduces session volume and duration.

Experiments on practicing the strength test have been conducted using maximal isometric exercise [i.e., isometric maximal voluntary contraction (MVC) training] and maximal loads during coupled eccentric–concentric repetitions [i.e., one repetition maximum (1RM) training]. In some interventions, the strength test has been practiced daily. In other interventions, the strength test has been practiced on nonconsecutive days. Strength tests that are practiced daily can be considered a type of resistance exercise snacks.

### Practicing Maximal Isometric Exercise

Performing maximal contractions regularly for exercise and rehabilitation purposes is a concept that has been understood for several decades. Between 1894 and 1979, 34% of all research papers on resistance exercise included interventions with frequencies of 5, 6, or 7 days per week—called “daily training” [51]. These interventions often included low volumes of maximal isometric exercise of the elbow flexor, knee extensor, and hand grip muscles (Table 8). When performed daily for brief periods, practicing the strength test is a resistance exercise snack with maximal resistance. As shown in Table 8, daily maximal isometric exercise increases isometric MVC strength. Though performing the same isometric tasks at submaximal resistances is also likely to increase muscle strength and endurance [84–87], we have highlighted only interventions that involve practicing maximal isometric strength tests, in part, because these would be the most time efficient. The isometric MVC requires only a couple of seconds of effort to recruit the entire motor neuron pool, whereas submaximal isometric contractions require more time to achieve the same physiological outcome [88]. The study by Cotten [89] serves as a useful example. In this study, four groups of participants completed a single set involving one sustained isometric contraction until failure. One group performed an isometric MVC and the other groups performed the task until failure at 25, 50, and 75% MVC. The four groups each improved their isometric strength by ~ 10%. Thus, performing the brief MVC was the most time efficient way to achieve the 10% increase in muscle strength.

**Table 8:** Practicing the isometric strength test—summary of results from studies that examined changes in muscle strength after isometric resistance exercise programs that typically involved five to seven sessions per week, completed in 3 min or fewer per session, and involved minimal numbers of repetitions at maximal repetitions [i.e., 100% of the isometric maximal voluntary contraction (MVC)]

Reference	Group	Sample				Session					Week			Program	
		RE past	*n*	Sex	Age (years)	Exercise no.	Set no.	Rep no.	Intensity (%max)	Time (min)	FREQ (days/week)	Set no.	Rep no.	Duration (weeks)	Strength (% $\Delta$)
Vanderhoof et al. [191]	EX1	NR	5	M	NR	1 grip	1	1 (6 s)	100	< 1	5	5	5	29	42^a^
	EX2	NR	5	M	NR	1 grip	1	Fail	100	~ 2	5	5	5	29	58^a^
	CON	NR	5	M	NR	0	0	0	0	0	0	0	0	29	2^a^
Byrd and Hills [192]	EX1	NR	6	M	31	1 grip	1	1 (fail)	100	~ 1.5	5	5	5	4	14^a^
	CON	n/a	n/a	n/a	n/a	n/a	n/a	n/a	n/a	n/a	n/a	n/a	n/a	n/a	n/a
Walters et al. [193]	EX1	NR	6	MW	25	1 EF	1	1 (15 s)	100	< 1	5	5	5	1.5	16^a^
	CON	n/a	n/a	n/a	n/a	n/a	n/a	n/a	n/a	n/a	n/a	n/a	n/a	n/a	n/a
Mathews and Kruse [194]	EX1	NR	15	M	NR	1 EF	1	3 (6 s)	100	< 1	5	5	15	4	~ 10^a^
	CON	n/a	n/a	n/a	n/a	n/a	n/a	n/a	n/a	n/a	n/a	n/a	n/a	n/a	n/a
Rasch and Pierson [195]	EX1	NR	29	M	NR	1 EF	3	1 (15 s)	100	~ 3	5	15	15	5	17^a^
	CON	n/a	n/a	n/a	n/a	n/a	n/a	n/a	n/a	n/a	n/a	n/a	n/a	n/a	n/a
Rasch and Pierson [196]	EX1	NR	14	M	NR	1 EF	3	1 (15 s)	100	~ 3	5	15	15	5	12^a^
	CON	n/a	n/a	n/a	n/a	n/a	n/a	n/a	n/a	n/a	n/a	n/a	n/a	n/a	n/a
Cotten [89]	EX1	NR	6	MW	NR	1 EF	1	1 (fail)	25	NR	5	5	5	NR	8^a^
	EX2	NR	6	MW	NR	1 EF	1	1 (fail)	50	NR	5	5	5	NR	13^a^
	EX3	NR	6	MW	NR	1 EF	1	1 (fail)	75	NR	5	5	5	NR	11^a^
	EX4	NR	6	MW	NR	1 EF	1	1	100	NR	5	15	15	NR	13^a^
	CON	n/a	n/a	n/a	n/a	n/a	n/a	n/a	n/a	n/a	n/a	n/a	n/a	n/a	n/a
Friedebold et al. [197]	EX1	NR	12	W	19–21	1 KE	1	1 (10 s)	100	< 1	5	5	5	10	82^a^
	CON	n/a	n/a	n/a	n/a	n/a	n/a	n/a	n/a	n/a	n/a	n/a	n/a	n/a	n/a
Ikai and Fukunaga [198]	EX1	NR	5	M	23–28	1 EF	3	1 (10 s)	100	~ 2.5	6	18	18	14	92^a^
	CON	n/a	n/a	n/a	n/a	n/a	n/a	n/a	n/a	n/a	n/a	n/a	n/a	n/a	n/a
Grimby et al. [199]	EX1	NR	20	W	19–23	1 EE	1	30 (3 s)	100	3	5	5	150	6	32^a^
	CON	NR	20	W	19–23	0	0	0	0	0	0	0	0	0	5^a^
Lucca and Recchiuti [200]	EX1	NR	20	W	20	1 KE	1	5 (25 s)	100	NR	5	5	25	4	0–18^a^
	CON	NR	10	W	20	0	0	0	0	0	0	0	0	n/a	− 7^a^
Szeto et al. [201]	EX1	No	6	MW	NR	1 KE	3	10 (5 s)	25	7	5	15	150	3	22^a^
	EX2	No	6	MW	NR	1 KE	3	10 (5 s)	50	7	5	15	150	3	31^a^
	EX3	No	18	MW	NR	1 KE	3	10(5-s)	100	7	5	15	150	3	46^a^
	CON	n/a	n/a	n/a	n/a	n/a	n/a	n/a	n/a	n/a	n/a	n/a	n/a	n/a	n/a
Barss et al. [98]	EX1	Yes	8	MW	~ 24	1 grip	5	1(3-s)	100	NR	7	35	35	2.5	10^a^
	CON	n/a	n/a	n/a	n/a	n/a	n/a	n/a	n/a	n/a	n/a	n/a	n/a	n/a	n/a

Other outcomes that improved in some of the above studies after practicing the strength test exercise included: muscle endurance [89, 191–193, 199]. No change in muscle size was observed in one study [195]

*CON* control group (no exercise), *EF* elbow flexion, *EX* exercise group, *FREQ* frequency, *KE* knee extension, *M* men, *n/a* not applicable, *NR* not reported, *RE* resistance exercise, *W* women

^a^Isometric strength test

^b^One repetition maximum concentric strength test

Studies on daily maximal grip training can be considered proof of concept for the broader ideas of practicing the strength test and of minimal resistance exercise doses. However, daily maximal grip practice should not be disregarded as its own standalone exercise prescription (i.e., ecological validity), particularly in rehabilitation programs for patients whose grip capacity is reduced (e.g., stroke). Grip strength correlates with mortality [90–92] and ability to perform activities of daily living [93–96]. Moreover, one review concluded that isometric hand grip exercise, which is often minimal in its prescription, reduces resting systolic blood pressure and induces hypoalgesia [97]. Also, the ability to perform such exercise on consecutive days could lessen injury recovery times. For example, in a group of healthy participants who had resistance exercise experience, Barss et al. [98] compared maximal hand grip training for 18 consecutive days versus a more traditional prescription that spread maximal hand grip training over 42 days but still totaled 18 sessions (i.e., three sessions per week for 6 weeks). The traditional program increased peak force of the trained and untrained hands by 14.6 and 12.5%, respectively. The trained and untrained limbs became significantly stronger at the end of the third and fourth weeks of training, respectively. The daily grip training increased peak force in the trained and untrained hands by 9.7 and 7.8%, respectively. The untrained limb became significantly stronger after the 15th day of training. Thus, the study illustrated that if the purpose of a rehabilitation program is to significantly increase grip strength in the untrained limb as quickly as possible, then daily maximum grip training is the more appropriate approach.

### Practicing Eccentric–Concentric Repetitions with Maximal Loads

Using the daily 1RM training model, Dankel et al. [99] examined changes in muscle size and strength of the elbow flexors in five resistance-trained men who completed different resistance exercise programs with their right and left arms for 21 consecutive days. With one arm (“training arm”), they performed a 1RM test, an isometric MVC, and three sets of exercise at 70% 1RM. With their other arm (“testing arm”), participants completed only the 1RM and MVC strength tests. After 21 consecutive days, 1RM strength increased similarly (~ 2 kg) in the “training arm” and “testing arm,” whereas muscle thickness increases occurred only in the “training arm.” Also, no improvements in MVC strength were observed in either group.

Using the nonconsecutive days 1RM training model in untrained participants, Mattocks et al. [100] compared practicing the strength test to a higher exercise volume protocol. One group of participants completed 1RMs for the knee extension and chest press exercises 2 days per week for 8 weeks, while another group completed four sets of those exercises to volitional failure with an 8–12RM load. Overall, gains in muscle strength 1RM, isometric, and isokinetic strength were similar between the two groups. Improvements in upper-body muscle endurance (repetitions to failure at 60% 1RM) were also similar between groups. However, the group that completed a greater volume of exercise experienced more muscle hypertrophy of the triceps brachii and vastus lateralis, and greater improvements in muscle endurance of the knee extensors.

In another study [41], 20 untrained young adults completed two resistance exercise sessions per week for 8 weeks. Each session consisted of five repetitions of both the chest press and knee extension machine exercises. Participants were assigned the task of attempting to lift the maximal resistance possible for each repetition, with 90-s rest between repetitions. Participants who completed this minimal dose program experienced affective responses during the exercise sessions (e.g., revitalization and positive engagement) that were equal to or slightly better than the responses experienced among a separate group of participants who completed four sets of 8–12 repetitions (to volitional failure) per exercise session. Neither exercise program improved self-efficacy.

Overall, practicing the isometric MVC test daily over a few weeks improves isometric muscle strength. Thus, maximal isometric contractions can be prescribed as minimal doses for individuals who do not participate in resistance exercise and who find eccentric–concentric resistance exercise unfeasible or unenjoyable. Moreover, daily minimal doses of maximal isometric contractions might represent an underutilized strategy for increasing muscle strength quickly. Preliminary results from research on practicing the 1RM strength test on nonconsecutive days appear promising [100], but more research is required to determine whether this prescription method is feasible and whether it increases muscle strength in untrained individuals in nonlaboratory settings.

## Minimal Doses of Eccentric Resistance Exercise

Muscle action or contraction type is one variable of resistance exercise programming that has received little attention in position papers on resistance exercise guidelines (Table 1) and in previous discussions on minimal dose resistance exercise strategies [47]. A typical repetition of a resistance exercise consists of both an eccentric (i.e., active muscle lengthening) and concentric (i.e., active muscle shortening) muscle action (i.e., a coupled eccentric–concentric repetition).

Eccentric resistance exercise has been known for many years to provide a potent stimulus for improving muscle size and strength [101–105]. Eccentric resistance exercise also increases joint range of motion [106–108] and thus can replace static stretching in an exercise program to reduce overall exercise time [109]. Also, when eccentric and concentric resistance exercise are completed with equal absolute workloads, cardiovascular stress and perceptions of effort are lower during eccentric exercise [110–115]. This suggests a unique role for prescriptions of eccentric resistance exercise for older adults and those with cardiovascular or other medical conditions [116, 117], who might benefit from minimal dose training strategies. Nevertheless, we are aware of only one paper that has discussed eccentrics as a potential minimal dose approach [47].

A likely reason that eccentric resistance exercise has not received more attention in previous discussions on minimal dose strategies is that traditional resistance exercise equipment (e.g., free weights, plate-loaded machines, and weight stack machines) is not conducive to performing eccentric resistance exercise [118]. Exercising with such equipment involves use of the same load in the eccentric and concentric phases, which probably does not maximize dose potency. The reason that exercise dose potency is likely hindered is that concentric muscle strength is ~ 40% less than eccentric muscle strength in humans [53], and traditional resistance exercise equipment necessarily accommodates the weaker concentric phase. As traditional equipment is what most individuals have access to, there has not been a need to consider muscle contraction type in resistance exercise guidelines. Such guidelines have always assumed eccentric–concentric repetitions performed with a given constant external load. However, these assumptions require reconsideration because new exercise equipment is making differential loading in the eccentric and concentric phases possible [119–122]. Consequently, opportunities to participate in eccentric-only and accentuated eccentric resistance exercise (i.e., “eccentric overload”) are likely to increase in the future.

Minimal dose eccentric resistance exercise involves a low weekly session volume of submaximal or maximal eccentric-only repetitions. Variables of the exercise prescription that are minimized with minimal dose eccentric prescriptions are the number of concentric (i.e., zero) and eccentric muscle actions. Minimal dose eccentric exercise performed with maximal eccentric resistances [123–125] represents a specific type of practicing the strength test or maximal resistance exercise snacking. Research on minimal dose eccentrics is currently at the proof-of-concept stage.

In 1960, Bonde-Petersen [123] examined the effects of ten daily maximal eccentric repetitions, ten daily isometric MVCs, and one daily isometric MVCs on muscle strength and found that MVC strength increased only for participants who completed ten daily isometric MVCs. However, results from contemporary studies challenge these original findings (Table 9).

**Table 9:** Eccentric minimal dose resistance exercise—summary of results from studies that have examined changes in muscle strength after minimal dose resistance exercise programs comprised of eccentric-only repetitions

Reference	Group	Sample				Session					Week			Program	
		RE past	*n*	Sex	Age (years)	Exercise no.	Set no.	Rep no.	Intensity (%max)	Time (min)	FREQ (days/eeek)	Set no.	Rep no.	Duration (weeks)	Strength (% $\Delta$)
Bonde Petersen [123]	EX1	NR	6	MW	~ 25	1 EF	1	10	100	NR	5	5	50	8	5–9^a^
	CON	NR	13	MW	~ 25	0	0	0	0	0	0	0	0	8	− 8 to2^a^
Chen et al. [127]	EX1	No	13	M	21	1 EF	5	6	10	NR	2	10	60	4	28–71^ac^
	CON	No	n/a	n/a	n/a	n/a	n/a	n/a	n/a	n/a	n/a	n/a	n/a	n/a	n/a
Crane et al. [202]	EX1	Yes	15	MW	23	1 LP	2	2 min	50–75	6	1	2	NR	6	27^b^
	EX2	Yes	15	MW	23	1 LP	2	2 min	50–75	6	3	6	NR	6	37^b^
Duncan et al. [203]	EX1	No	16	M	24	1 KE	1	3	100	NR	3	3	9	8	1–34^bc^
	CON	No	18	M	24	0	0	0	0	0	0	0	0	8	− 5 to 1^bc^
Sato et al. [124]	EX1	No	13	MW	21	1 EF	1	7	100	NR	7	7	49	3	10–13^abc^
	CON	No	10	MW	21	0	0	0	0	0	0	0	0	3	− 5 to 1^abc^
Yoshida et al. [125]	EX1	No	12	MW	21	1 EF	1	6	100	NR	1	1	6	4	0^a,b,c^
	EX2	No	12	MW	21	1 EF	1	6	100	NR	5	5	30	4	8–14^abc^
	EX3	No	12	MW	21	1 EF	5	6	100	NR	1	5	30	4	0^a,b,c^
	CON	n/a	n/a	n/a	n/a	n/a	n/a	n/a	n/a	n/a	n/a	n/a	n/a	n/a	n/a
Yoshida et al. [126]	EX1	No	13	MW	22	1 EF	1	1	100	6 s	2	2	2	4	0
	EX2	No	13	MW	22	1 EF	1	1	100	6 s	3	3	3	4	0–5^abc^
	EX3	No	13	MW	21	1 EF	1	1	100	6 s	5	5	5	4	10–15^abc^
Johnson et al. [204]	EX1	NR	14	MW	64	1 RS	1	23 steps/min	20–50% MES	3–10	2	2	n/a	8	~ 35^b^
	CON	n/a	n/a	n/a	n/a	n/a	n/a	n/a	n/a	n/a	n/a	n/a	n/a	n/a	n/a
Kay et al. [205]	EX1	NR	12	MW	~ 67	1 RS	1	40 steps/min	50	5–10	2	2	n/a	6	59^b^
	EX2	NR	15	MW	~ 70	RS + PF	1	40 steps/min	50	RS: 5–10 PF: 5	2	2	n/a	6	39^b^

Other outcomes that improved in some of the above studies after minimal dose eccentric resistance exercise included: muscle size [125], muscle strength [204, 205], vertical jump height [202], balance [204]. No changes in muscle size were observed in other studies [124, 126]. Timed up-and-go increased and was retained after 8 weeks of detraining [205]

*CON* control group (no exercise), *EF* elbow flexion, *EX* exercise group, *FREQ* frequency, *LP* leg press, *KE* knee extension, *KF* knee flexion, *M* men, *MES* maximal eccentric strength, *n/a* not applicable, *NR* not reported, *PF* plantar flexion, *RE* resistance exercise, *RS* recumbent stepper, *W* women

^a^Isometric strength test

^b^Eccentric strength test

^c^Concentric strength test

Sato et al. [124] compared the effects of a 3-s isometric MVC, concentric MVC, or eccentric MVC of the elbow flexors performed daily (5 days per week for 4 weeks) on muscle strength of the elbow flexors and muscle thickness of biceps brachii and brachialis. Exercise was performed on an isokinetic dynamometer. Participants who performed the once daily eccentric MVC had the most robust improvements in muscle strength. Across isometric, concentric, and eccentric MVC strength tests, the group that performed the minimal dose eccentric exercise improved their muscle strength by 10–13%. The group who performed the once daily concentric MVC improved only their isometric MVC torque by 6%. The group who performed the once daily isometric MVC improved only their eccentric MVC torque by 7%. The exercise protocols caused little to no muscle soreness and no changes in muscle thicknesses. The control group showed no changes in muscle strength.

Yoshida et al. [125] compared the effects of different minimal dose maximal eccentric resistance exercise programs of the elbow flexors on muscle strength of the elbow flexors and muscle thickness of biceps brachii and brachialis. Thirty-six healthy university students were randomized into three groups who performed the exercise on an isokinetic dynamometer: 1 day per week (one set × six maximal eccentric contractions, six contractions per week), 1 day per week (five sets × 6 maximal eccentric contractions once per week, 30 contractions per week), or 5 days per week (one set × 6 maximal eccentric contractions, 30 contractions per week). The two groups who performed only 1 day of exercise per week did not experience changes in muscle strength. The group who performed the exercise 5 days per week increased eccentric MVC torque (13.5%), concentric MVC torque (11.1%), and isometric MVC torque (9.3%). The exercise protocols caused little or no muscle soreness. The results indicated that completing a small number of maximal eccentric contractions throughout the week leads to greater gains in muscle strength than performing a larger volume of eccentric muscle actions once per week. In a follow-up study, Yoshida et al. [126] found that one 3-s maximal eccentric contraction of the elbow flexors improved muscle strength by 10–15% when performed once per day, 5 days per week, over 4 weeks. However, participants who performed the same exercise three days per week experienced only a ~ 3% increase in strength, and participants who performed the exercise once per week experienced no change in muscle strength. None of the groups experienced muscle hypertrophy.

Chen et al. [127] examined the effects of repeating 30 low-load eccentric muscle actions on muscle strength of the elbow flexors and muscle thickness of biceps brachii and brachialis. The dumbbell used in the study was equal to 10% of the isometric MVC. The study included three groups of participants who performed eccentric exercise in different configurations: 1 bout, 8 bouts (2 bouts per week for 4 weeks), or 16 bouts (2 bouts per week for 8 weeks). The results indicated that repeating low-intensity eccentric resistance exercise increased muscle size and strength and protected against future muscle damage of the exercised muscles.

Overall, results from studies summarized in this section and in Table 9 suggest that minimal dose eccentrics can be advocated as a minimal dose approach for those who are currently not participating in resistance exercise. Also, although the current review is focused primarily on nonathlete populations, it is important to acknowledge that the Nordic hamstring exercise is another eccentric-only exercise that has been prescribed in low weekly volumes and been found to increase muscle strength in competitive and recreational athletes in most instances [128–132].

## Strategies to Enhance Minimal Doses

In the preceding sections, we illustrated that various minimal dose approaches to resistance exercise increase muscle strength. We acknowledge that greater gains in muscle size and strength are possible with higher exercise volumes and intensities [72, 133, 134]. Thus, methods to increase exercise volume while maintaining the same minimal exercise time warrant discussion.

Drop sets are a method that can be used to prolong time under tension. Drop sets involve performing a set of resistance exercise to momentary muscular failure (or close to failure), then immediately reducing the load (multiple times) to increase the work completed over a brief time. In untrained and trained men, drop sets provided similar gains in muscle size and strength as volume-matched routines without drop sets [135–137]. Moreover, new connected adaptive resistance exercise machines (CARE) make drop sets more feasible than with traditional resistance exercise equipment [118–121]. Unlike with free weights, where the individual must momentarily disengage with the resistance to remove bar collars and weight plates to perform the next lighter drop set, CARE machines automatically reduce the resistance for the individual.

Rest–pause training is another strategy that can be used to enhance minimal resistance exercise doses. Rest–pause training involves lifting a fixed load with an initial set to failure (typically 10–12 repetitions), followed by subsequent sets to failure using short (e.g., 10–20 s) interset rest intervals [138]. For instance, 20 repetitions might be achieved by first completing 12 repetitions, followed by 4, then 3, then 1 repetition interspersed by short 20-s rest periods. Rest–pause training performed over several weeks causes comparable or larger increases in muscle size and strength compared with resistance exercise routines that do not involve rest–pauses in trained individuals [139–141]. Importantly, rest–pause training reduces exercise session duration. For instance, in one study, session time was reduced from ~ 57 min during traditional resistance exercise sessions to ~ 35 min during sessions that incorporated rest–pauses [141]. Thus, rest–pause resistance exercise appears to provide the same benefits as traditional resistance exercise strategies while reducing exercise time.

Nevertheless, drop set and rest–pause strategies exacerbate acute muscle fatigue and increase perceptions of discomfort and fatigue compared with more traditional resistance exercise methods, where sets are not taken to failure [142, 143]. Thus, drop set and rest–pause strategies might not be feasible for individuals who have low pain tolerances or who are currently not participating in any resistance exercise. Instead, drop set and rest–pause strategies might be more appropriate for, and of greater interest to, individuals who are seeking to add variety to their current minimal dose programs.

## Preferences for Resistance Exercise

Preferences for resistance exercise, a topic that has been minimally researched [27], should also be considered when prescribing minimal doses of resistance exercise. Preferences often exist for exercise location (home, gym, outdoors, and treatment center), interpersonal contact during exercise (exercise alone, exercise with friend, and exercise with group), supervision, competition, equipment type (free weights, elastic bands, etc.), and exercise intensity (low, moderate, or high) [27]. Preferences might also exist for aspects of the resistance exercise experience closely linked to minimal dose prescriptions, for example, preferred weekly training frequency, session duration, and set configurations. Based on the evidence overviewed herein, individuals can, for the most part, choose the dosing strategy that meets their personal preferences and schedules. “Weekend Warrior”, single-set resistance exercise, and resistance exercise snack approaches are probably the minimal dose approaches that offer the best combination of practicality and potential for increasing muscle strength.

## Limitations of Minimal Dose Approaches

The most notable limitation of minimal dose approaches is that they are unlikely to induce the same magnitude of improvement in physical and mental health outcomes compared with more traditional approaches to resistance exercise. For example, individuals who exercise more than 1 day per week (not exclusive to resistance exercise) are further protected from mortality than “Weekend Warriors” [56, 58, 60]. Also, greater resistance exercise frequencies cause the greatest improvements in muscle size and strength because they entail greater exercise volumes [62–64]. Moreover, multiset resistance exercise programs typically cause greater improvements in muscle size and strength than single-set programs when the two are not matched for exercise volume [69–72]. Nevertheless, as shown in the current review, minimal dose approaches increase muscle strength and some other fitness outcomes, and this is more than what occurs with no resistance exercise. Minimal dose approaches might also act as “gateways” to more traditional resistance exercise programs, whereby initial exposure to, and adaptation from, a minimal dose program might cause longer-term behavior change.

A second potential limitation of some minimal dose approaches, such as daily exercise snacking, is that some individuals will not be able to adopt such a program due to lack of access to resistance exercise equipment at home or at work. Nevertheless, some resistance exercise equipment is accessible and affordable (e.g., elastic bands). Also, there are many instances in which access to resistance exercise equipment is a short distance from one’s residence. For example, fitness centers are located on school campuses where many students and staff reside and work; living residences (e.g., apartment complexes, hotels) often have small fitness centers within them; and some workplaces (11–18%) have fitness centers or equipment onsite [144, 145]. Local parks might also have equipment for body weight exercises such as push-ups, pull-ups, and step-ups. Thus, access to resistance exercise equipment might not be a significant barrier to some minimal dose strategies.

A third potential limitation is that in some studies cited in the tables, interventions were comprised of only one single-joint exercise. For example, studies on minimal dose eccentric resistance exercise have often consisted of only unilateral elbow flexion exercise on an isokinetic dynamometer [124, 125, 146]. Such studies lack direct practical application because isokinetic dynamometers are not readily available to most individuals, and exercise programs should target more than one muscle group of one limb. These studies, then, provide proof of concept of the minimal dose approach, but they lack ecological validity. Moreover, muscle soreness and damage from eccentric resistance exercise is another potential concern, though the minimal eccentric dose strategies reviewed here caused little or no muscle damage and soreness [124, 125]. New technologies are making eccentric resistance exercise, including multijoint exercise, more feasible outside laboratory environments [118, 122, 147]. Thus, individuals undertaking eccentric resistance exercise at home or the gym should be made aware of the possibility of muscle damage and how to minimize it. Future research can continue to explore the dose–response relationship between eccentric resistance exercise and muscle damage. This research can seek to establish eccentric resistance exercise dose–response relationships for various muscle groups and explore ways to prescribe eccentric resistance exercise that minimize exercise-induced muscle damage while causing gains in muscle size and strength. This can lead to recommendations on how to best periodize or progress eccentric resistance exercise.

A fourth potential limitation is that some of the cited studies involved direct instruction and supervision and participation under controlled conditions. Supervision and verbal encouragement impact muscle strength performance and outcomes from resistance exercise interventions [1, 148, 149]. Thus, effects observed in some of the cited studies might be larger than what would be expected in non-research settings. Moreover, in some of the studies cited in this review, encouragement and supervision occurred within the context of interventions that involved maximal efforts and/or use of maximal or near-maximal resistances (e.g., practicing the strength test). Individuals who undertake resistance exercise unsupervised are unlikely to exercise with maximal or near-maximal resistances [27, 150]. This then questions the ecological validity or feasibility of minimal dose programs that involve maximal contractions. Nevertheless, even if individuals were prescribed a program of maximal isometric contractions, and their actual effort amounted to 70 or 80% MVC, the strength of their muscles is still likely to increase [86].

Finally, we have not provided a recommendation for which minimal dose programs should be adopted. Instead, we have shown evidence that various minimal dose approaches improve or maintain muscle strength in nonathlete populations. Thus, individuals who are currently not partaking in resistance exercise should be encouraged to participate in the resistance exercise program that they are most likely to adhere to over an extended period. Future research can help to describe dose–response relationships for various minimal dose approaches and determine which minimal dose programs result in the greatest exercise adherence and/or health benefits. The current review focused on muscle strength as a key outcome, because muscle strength correlates with mortality and other outcomes of health and fitness [1–3]. The results presented in the table footnotes throughout the current paper show that minimal dose approaches sometimes improve outcomes other than muscle strength, such as muscle mass, muscle endurance, and up-and-go times. The effectiveness of minimal dose approaches for improving other outcomes, such as risk of falls, can be considered in future research.

## Conclusions

Minimal dose approaches to resistance exercise increase muscle strength and sometimes improve other physical fitness outcomes. Thus, even though minimal dose approaches might not meet current guidelines for resistance exercise published by professional exercise organizations, adoption of minimal dose approaches can be encouraged for individuals who do not engage in any resistance exercise. Such individuals can be informed that “something is better than nothing” and “every muscle contraction (or repetition) counts.” Individuals who have the time and resources to participate in exercise volumes that are greater than minimal dose programs should be encouraged to do so. Future research can explore dose–response relationships of minimal and traditional approaches to resistance exercise to determine their associated adherence rates and health benefits.

## Declarations

### Data and code availability

There are no data associated with this manuscript.
